# Unveiling the Chemistry of Citrus Peel: Insights into Nutraceutical Potential and Therapeutic Applications

**DOI:** 10.3390/foods13111681

**Published:** 2024-05-27

**Authors:** Hussan Munir, Sanabil Yaqoob, Kanza Aziz Awan, Aysha Imtiaz, Hiba Naveed, Naveed Ahmad, Muhammad Naeem, Waleed Sultan, Yongkun Ma

**Affiliations:** 1 School of Food and Biological Engineering, Jiangsu University, Zhenjiang 212013, China; hussan.munir@gmail.com (H.M.); sanabily67@gmail.com (S.Y.); 2 University Institute of Food Science and Technology, University of Lahore, Lahore 54590, Pakistan; 3 Department of Food Science and Technology, Faculty of Science and Technology, University of Central Punjab, Lahore 54000, Pakistan; kanza.aziz@ucp.edu.pk (K.A.A.); hibanaveed9764@gmail.com (H.N.); waleed.sultan@ucp.edu.pk (W.S.); 4 National Institute of Food Science and Technology, University of Agriculture, Faisalabad 03802, Pakistan; ayesha.ak124@gmail.com; 5 Joint Center for Single Cell Biology, Shanghai Collaborative Innovation Center of Agri-Seeds, School of Agriculture and Biology, Shanghai Jiao Tong University, Shanghai 200240, China; naveedjlau@gmail.com; 6 Department of Plant Science, School of Agriculture and Biology, Shanghai Jiao Tong University, Shanghai 200240, China; naeembbt@gmail.com

**Keywords:** citrus peel, extraction, supercritical, hyperlipidemia, hypoglycemic, antidiabetic

## Abstract

The recent millennium has witnessed a notable shift in consumer focus towards natural products for addressing lifestyle-related disorders, driven by their safety and cost-effectiveness. Nutraceuticals and functional foods play an imperative role by meeting nutritional needs and offering medicinal benefits. With increased scientific knowledge and awareness, the significance of a healthy lifestyle, including diet, in reducing disease risk is widely acknowledged, facilitating access to a diverse and safer diet for longevity. Plant-based foods rich in phytochemicals are increasingly popular and effectively utilized in disease management. Agricultural waste from plant-based foods is being recognized as a valuable source of nutraceuticals for dietary interventions. Citrus peels, known for their diverse flavonoids, are emerging as a promising health-promoting ingredient. Globally, citrus production yields approximately 15 million tons of by-products annually, highlighting the substantial potential for utilizing citrus waste in phyto-therapeutic and nutraceutical applications. Citrus peels are a rich source of flavonoids, with concentrations ranging from 2.5 to 5.5 g/100 g dry weight, depending on the citrus variety. The most abundant flavonoids in citrus peel include hesperidin and naringin, as well as essential oils rich in monoterpenes like limonene. The peel extracts exhibit high antioxidant capacity, with DPPH radical scavenging activities ranging from 70 to 90%, comparable to synthetic antioxidants like BHA and BHT. Additionally, the flavonoids present in citrus peel have been found to have antioxidant properties, which can help reduce oxidative stress by 30% and cardiovascular disease by 25%. Potent anti-inflammatory effects have also been demonstrated, reducing inflammatory markers such as IL-6 and TNF-α by up to 40% in cell culture studies. These findings highlight the potential of citrus peel as a valuable source of nutraceuticals in diet-based therapies.

## 1. Nutraceuticals and Pharma Foods

In dietary regimen, functional and nutraceutical foods are gaining immense importance due to their health-enhancing potential [1,2]. These foods go beyond basic nutrition to provide additional health benefits that can help prevent disease, thereby promoting overall health and well-being [3]. In spite of modern medical technologies, dietary interventions are gaining more popularity, owing to their long-term administration consistency and antagonizing effect of various pharmaceuticals [4]. In this regard, functional and nutraceutical foods are considered as a more cautious and sustainable approach for health management [5]. Internationally, consumers have great concern for using natural resources as an adjunct to pharmaceuticals against many ailments [6]. The thought of food as a therapeutic agent can be traced back up to the era of Hippocrates, the father of medicine [7].

Functional foods may be natural or with certain components added or removed as per their effectiveness, technological, or biotechnological practices [8]. Moreover, a food with an altered nature of its one or more constituents, or one in which the bioavailability of one or more of its components has been customized, or any combination of these possibilities, also comes under the term functional foods, nutraceuticals, and designer and therapeutic foods [9]. In addition, these foods contain medicinal properties and are found in the prophylaxis of certain diseases such as cardiac events, hepatic and renal disparities, hyperlipidemia, cancer, and PCOS; they also possess certain bio-regulatory functions [10,11,12,13].

A diet rich in phytochemicals or bioactive components is of choice for the present community because it is responsible for a decline in mortality rates due to their disease defensive mechanism [14]. People consuming functional and nutraceutical diets are less prone to illnesses, so they enjoy healthier lives [15]. Moreover, the outcome of lifestyle-related disparities has been augmented due to an unhealthy lifestyle, poor eating habits, and consumption of junk food. Moreover, a sedentary lifestyle and lack of exercise is also a major contributor of these ailments [16].

Bioactive or functional components of plant-based foods have been claimed to have disease-combating activities, and their regular utilization improves health status [17,18]. Recent advancements in the field of food and nutrition have focused on the consumption of functional foods, and consuming functional foods will become the largest adaptive trend in coming decades [19,20]. The health-enhancing and disease-preventive activities of these foods are due to some non-nutritive or bioactive components [21]. These bioactive components are different for different foods and include catechins, flavonoids, polyphenols, anthocyanin, carotenoids, sulforaphane, isothiocyanates, fibers, and essential oils [22].

Poor dietary habits and sedentary lifestyles have created many health disparities like cardiovascular diseases, high blood pressure, diabetes, obesity, osteoporosis, and cancer, resulting in reduced life expectancy [23]. Advances in medical science have provided mankind different ways to cope with these perils. However, their safety is a matter of prime care. An increase in oxidative stress is the cardinal root for all of these diseases [24].

**Figure 1:**
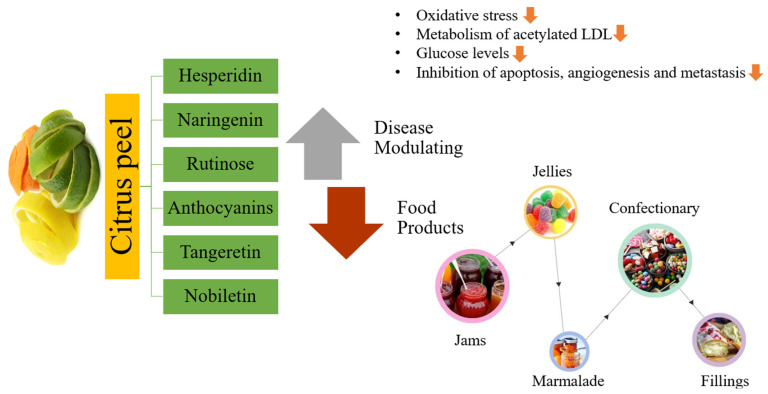
Exploring citrus peel bioactives: their role in health promotion and potential product applications.

Among agricultural waste materials, the citrus peel has now become the latest issue for researchers, who are exploring its rich phytochemical profile [25]. Interestingly, citrus peel is considered as waste and is believed to adversely affect the cleanliness of urban areas. However, their exploitation in food will not only offer an impending cost-effective innovative generation therapeutics but also enhance the value of functional and nutraceutical food products [26]. Citrus peel has been valorized for its phenolic content, e.g., phenolic acid and flavonoids [27]. In the present decade, phenolic compounds of natural origin are more desired due to their strong antioxidant potential either in food systems or in living organisms for various maladies [28]. Therefore, the present chapter is an effort to highlight the citrus peel’s dietary components and bioactive ingredients that are being exploited as therapeutic agents (Figure 1) because they are more economical, effective, and practical to reduce the risks associated with life-threatening disorders [29].

## 2. Citrus Peel: An Insight

Citrus genus belongs to family *Rutaceae* and subfamily *Aurantioideae*. It is categorized as a fruit having juicy vesicles containing segments [30]. Chief marketable citrus species comprise of orange (*Citrus sinensis*), grapefruit (*Citrus paradisi*), lemon (*Citrus limon*), lime (*Citrus aurantifolia*), and mandarin (*Citrus reticulata*) [31]. Citrus outer peel, having four portions, includes endocarp, albedo, epidermis, and flavedo [30]. The flavedo portion of citrus peel has chromoplasts, which give an orange, green, or yellow color, and glands containing essential oil [32]. The inner portion, commonly known as the mesocarp of citrus, is called the albedo; it is rich in hemicellulose, pectin and cellulose [33]. The inner flesh, or endocarp, consists of carpels filled with compacted juice packets. Endocarp is primary source of juice having sugar, organic acids, and pigments. Seeds of citrus fruit are found adjacent to core in center of fruit [34].

According to data published by FAO in 2020, the annual production of citrus is 158,490,986 tons, and more than one-third have been processed in some of developing countries, while up to 70% have been used in production of juice and concentrate [29,35]. Pakistan produces 2.6 million tons of citrus annually and is ranked 12th amongst the citrus-producing countries [36]. During the development of the juice industry in 1950s, researchers developed a new method regarding the use of high volumes of rind produce in processing [37,38]. Juices and fresh citrus fruits are considered beneficial for health [39]. Nevertheless, the main sources and benefits were not well known for many years. New health claims from citrus fruit and juice in physiological threats have been documented, including the investigation of properties of various health-enhancing compounds like phenolics, vitamin C, thiamine, folic acid, flavonoids, dietary fibers, carotenoids, and limionoids [40].

Citrus is a valued fruit in Pakistan, ranking first amongst all fruits in terms of land and output [41]. Pakistan’s environment is ideal for producing an extensive range of fruits. Citrus fruits account for almost 40% of the total amount of fruits harvested in Pakistan, where they are grown on an area of 199,400 hectares with a yearly production of approximately 2.29 million tons [42]. Punjab province produces more than 95% of citrus, with the Kinnow variety accounting for 70% of that total [43]. However, throughout the remaining regions of the globe, sweet orange varieties account for more than 70% of the market, owing to a wider range of types from early to late ripening and fewer seeds per fruit [44]. Pakistan’s average citrus production (11,000 kg/ha) is inadequate when contrasted to the standard yields in different citrus-producing countries like Brazil, the United States, and Turkey (22,000, 26,000, and 27,000 kg/hectare, correspondingly) [42,45]. The estimated annual yield of citrus throughout Pakistan is 18,000–20,000 kg/ha [42,46]. In Pakistan, the whole scenario of fruit cultivation is dominated by citrus with a large area and production [36]. Punjab accounts for 75% of the total production of citrus fruit in Pakistan [43,47]. About 33% of the total citrus fruit produced is processed commercially [48]. This percentage is greater in case of oranges, as more than 40% of globally produced oranges are utilized for processing [49]. The proportion of grapefruit utilization for processing is similar to that of oranges.

### Citrus Peel: Chemical Profile

Citrus peel is obtained as a by-product after juice extraction or any other citrus processing operation [50]. Pollution monitoring agencies and processing industries face severe problem due to the perishable nature of citrus peel [51]. In the present scenario, there is a need to utilize citrus peel to produce value added food products [52]. The utilization of by-products of fruit waste provides monitoring benefits and resolves environmental problems regarding citrus peel perishability [53]. Scientists have explored the rich phytochemistry of this agro base waste for the development of functional foods and as well as dietary supplements [54]. Consideration of people for the safe alternative of drugs also leads to the production of nutraceutical products using citrus peel [55]. The antibacterial property of citrus peel is also one of the leading causes to use it as a natural preservative instead of employing various synthetic preservatives in different food products [56,57] (Table 1).

Certain scientific approaches have explored such sources for coping with physiological threats that are cost-effective, easily assessable, and less toxic [61]. Fruits, vegetables, and their peels are cheap sources of bioactive components [62]. Citrus peel, a by-product of the juice processing industry, has proved its benefits due to its effectiveness against various diseases like cardiovascular complications, diabetes, oxidative stress, cancer, etc. [29]. These therapeutic effects are attributed to its flavonoid content, which has the power to scavenge free radicals and shield cells and protect tissues from degeneration [63,64].

Flavonoids are the largest group of naturally occurring phenolics that are mostly found in the citrus family [65]. Citrus peel is a rich source of polymethoxylated flavones and flavanones which are not present in other plants [66]. Two remarkable properties of citrus flavonoids are their bitter taste and low solubility, which maximize its recovery in polar solvents [67]. To explore the health-enhancing effects of peel extract, it is imperative to estimate the therapeutically active ingredients as well as to optimize its content in resultant extract [68].

The most prevalent flavanones present in orange peel (*Citrus sinensis*) are hesperidin, naringin, and rutinose, which make up to 6.5 g/L of essential oil [59]. Hesperetin and naringin are promising flavanones of orange peel and grapefruit peel, respectively [65]. Hesperidin is abundantly found in all over the peel, whereas naringin is predominant in the flavedo portion [69].

Polymethoxylated flavone (PMF) from orange peel is a mixture of 5.44% hydroxylated PMFs and 75.1% non-hydroxylated PMFs that show a comprehensive range of biological activity [70]. Polymethoxylated flavones such as nobiletin, sinenstin, and tangeretin are mostly confined in the essential oil of the flavedo portion; however, they are less frequent than flavonones [59]. Comparatively, they are common in citrus, as two polymethoxylated flavones (PMFs), nobiletin and tangeretin, are present in sweet and bitter orange peel [71].

**Figure 2:**
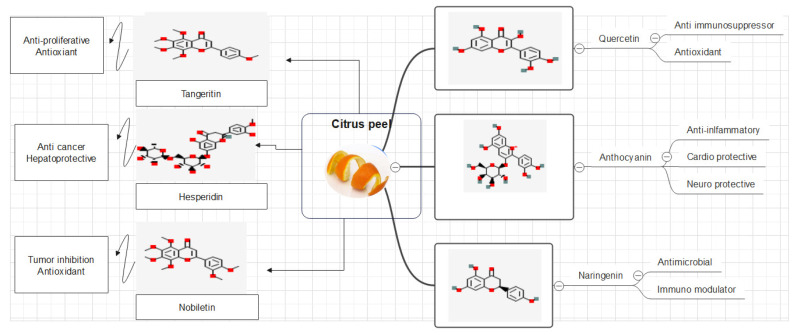
Biological properties of citrus peel bioactives.

In citrus fruits, two groups of flavonoids are present, including flavanone glycosides like hesperidin, naringin, and neohesperidin, and polymethoxylated flavones comprising nobiletin, tangeretin and sinensetin, as illustrated in Figure 2 [66]. Hesperidin, considered the best among all flavanone glycosides, is well known to reduce the permeability of capillaries and enhance vascular integrity; it is also used as supplement for fragility and for the permeability of blood vessel-compromised patients [72]. Hesperidin has certain pharmacological properties, like anti-inflammatory and analgesic effects, with oral carcinogenesis inhibitory effects and menopausal symptoms against estrogen-like effects [73].

Studies have shown the positive effects of nobiletin against Hepatitis-C, edema, sunburn (erythema), photo carcinogenesis, and photo aging [74]. About 1700 years ago, citrus was cultivated in China, and they believed that drugs and foods were from same source; citrus base ingredients are used commonly in TCM (Traditional Chinese Medicine) [75]. Menopausal symptoms and flatulence are cured in China using sour orange flower and mature whole citrus fruit, and the peel is also used in reducing lung phlegm. Immature citrus peel is recommended for digestion and gut disorders [76,77].

Biological and pharmacological properties of peel, such as antioxidant, anti-inflammatory, antiallergic, antiviral, antibacterial, antimutagenic and anticarcinogenic activities, are associated with these two major classes of flavonoids [65]. In the United States, previous studies have reported that the total intake of flavonoids should be 1 g/day for glycosides and 650 mg/day for aglycones. Keeping in view the health aspects associated with citrus peel flavonoids, food processors are engaged in the preparation of peel extracts of variable concentrations to be utilized as food supplements for humans [59,78]. For example, hesperidin that is extracted from de-oiled orange peel and contains both classes of flavonoids (flavanones and flavones) is being used in Western countries for various ailments [79].

## 3. Comparative Assessment of Citrus Peel Extraction Methods

Phenolic compounds are comprised of aromatic rings, and their enormous structural diversity ranges from simple to highly polymerized phenolics [2,80]. Phenolics naturally exist as conjugates of monosaccharaides, polysaccharides, and their derivatives, such as methyl esters [81]. They are categorized into three classes due to their structural divergence: phenolic acids, tannins, and flavonoids [80]. There are two subclasses of phenolic acids: hydroxycinnamic acids and hydroxybenzoic acids, which mostly occur in citrus by-products [82]. Hydroxycinnamic acids have aromatic ring structure, including caffeic, ferulic and sinapic acids, while hydroxylbenzoic acid comprises gallic, vanillic, syringic acids [83]. Tannins are high-molecular-weight compounds that are of less significance in the human diet [84].

**Figure 3:**
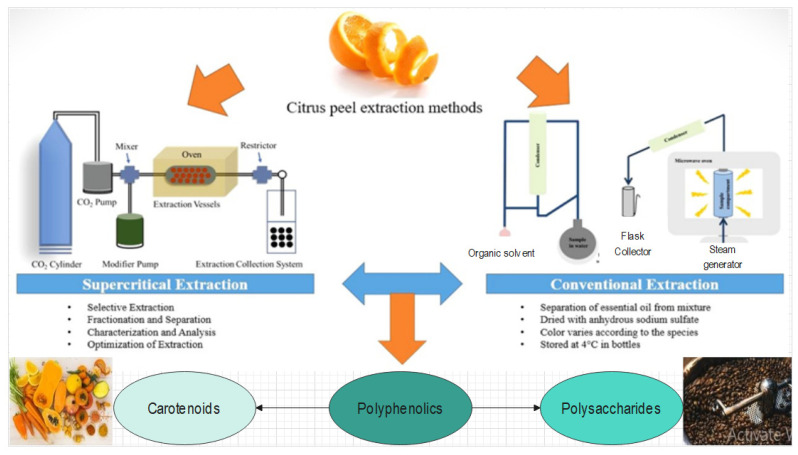
Extraction technologies employed for citrus peel phytoceutics.

Different solvents are used to extract bioactive components from citrus peel (Figure 3). Solvents such as ethyl acetate, petroleum ether acetone, ethanol, and water are used [85]. After lemon peel extraction, results have shown differences in the extraction yields of different solvents [86]. Ethyl acetate has the highest extraction yield (18%), followed by acetone, and ethanol has the least [87]. *Citrus sinensis* peel showed the maximum extraction yield in acetone (17%), followed by ethyl acetate, which was about 12% [88]. Aqueous extract has moderate extraction yield compared to other solvents [89].

Moreover, the supercritical fluid extraction of bioactive ingredients from natural compounds of different sources is widely investigated due to its immediate advantage on many other traditional extraction techniques [2,90,91]. Solvent selectivity and power make this process flexible, reducing the losses of expensive solvents and the polluting effect of organic solvents [92]. Many compounds are considered as supercritical fluid extraction solvents, including hydrocarbons such as butane, hexane, pentane, sulfur hexafluoride, nitrous oxide, and fluorinated hydrocarbons [93]. The most popular, safe, readily available, and low-cost solvent used in supercritical fluid extraction is carbon dioxide (CO_2_) [94]. It allows operations moderately at room temperature and different pressures [95]. The only drawback of supercritical fluid extraction is its high initial investment cost compared to conventional extraction techniques [96]. Nevertheless, the processing scheme is relatively cheap and simple [97].

In supercritical fluid extraction, fluids have better diffusion properties and lower viscosities [2,95]. The efficiency of supercritical fluid extraction (SFE) depends on the purity of the solvent, along with the supercritical fluid (CO_2_) and the motion of gases with the solvent [98]. The viscosity and density of these fluids exist between those of liquids and gases [99]. Likewise, the diffusivity of such fluids is aptly higher than that of liquids, which permits higher extraction rates [100]. Operating conditions like temperature and pressure are responsible for fluctuations in the extraction efficiency, enabling the regulation of the solvent effect [101]. Recently, supercritical fluid extraction (SFE) was considered as a good technique for extracting health-enhancing components [102].

A shorter extraction period and high selectivity in extraction compounds is offered by supercritical CO_2_ extraction, along with no solvent residue and with positive effects on extract quality [103]. The flexibility in controlling the variables intact in the supercritical fluids extraction process allows one to improve an experimental condition that considers the influence of a substance of interest [104,105]. Essential oil, carotenoids, fatty acids, phenolic compounds, and alkaloids have been extracted from various natural products using water at hydrothermal conditions and supercritical extraction [106].

In the field extraction of heat-sensitive components that are specially made for human nutrition, CO_2_ is the best supercritical fluid due to its physical and chemical properties and its values of low critical pressure and temperature [102]. Mainly in the fields of food application, water is also considered as a supercritical solvent for extraction [107]. Polar components are environmentally friendly, like water, especially for compounds sensitive to heat [108]. Extracts collected by the procedure of supercritical fluid extraction are of higher quality than those which are gained from steam and water distillation or from organic solvent extraction, which can induce water solubilization, hydrolysis, and the thermal degradation of some components [90,106].

## 4. Polyphenolic Moieties

Various chromatographic methods have been proven as safe techniques that provide more accurate, precise, and reproducible outcomes, such as column, thin-layer, gas, and liquid chromatography [109]. The application of each technology is dependent on the nature of the component to be detected and the sensitivity of the instrument [110]. In this respect, high-performance liquid chromatographic (HPLC) analysis is a crucial tool for the further characterization and quantification of biologically active ingredients of the resultant extract [111]. HPLC retention time and spectral data analysis of citrus peel bioactive ingredients confirmed the presence of C-glycosylated flavones, O-glycosylated flavones, polymethoxylated flavones, flavonol, O-glycosylated flavanones, and phenolic acids [112,113].

The presence of a specific ingredient in raw material depends on the type of solvent, temperature, and time [114]. Previously, it was documented that hesperidin was difficult to extract by hot water, while the contents of nobiletin were substantial [115]. Increased extraction temperature improved the yield of hesperidin but had little impact on narirutin, nobiletin, and tangeretin yield [116]. So, a considerable amount of PMFs can be extracted in water at a lower temperature, but the level of hesperidin is lower, even at a higher temperature [117].

Earlier, seven phenolic acids, including four hydroxycinnamics (caffeic, *p*-coumaric, sinapic, and ferulic) and three hydroxybenzoics (protocatechuic, *p*-hydroxybenzoic, and vanillic), were determined through HPLC [118]. The extraction temperature slightly influenced the phenolic acid concentration in extract [119]. Meanwhile, when increasing the extraction time (at 100 °C), individual phenolic acid decreased to some degree; it is supposed that some phenolic acids may be destroyed under hot water conditions with increases in time [120,121]. Another study revealed that under alkaline conditions, the hydrolysis of the bound form of phenolic acid from citrus peel increased at an ambient temperature [122].

Earlier, Sharma, Mahato [67] quantified the glycosylated flavanone and polymethoxylated flavones in mandarin peel using high-performance liquid chromatography with a photodiode array detector (HPLC-PDA). Results revealed that hesperidin (62.01 ± 0.24 mg/g dry peel weight) had the maximum concentration, followed by narirutin (7.66 ± 0.23 mg/g), nobiletin (0.31 ± 0.01 mg/g), and tangeretin (0.16 ± 0.22 mg/g dry peel weight), respectively. Afterward, [123] explored methanolic citrus peel extract for its antioxidant activity. Nobiletin (0.20 ± 0.01 mg/g) and hesperidin (0.5 ± 0.002 mg/g) were detected in citrus peel through the HPLC system; however, the tangerrtin concentration was low [124].

One of peel by-product, contain 85–99% volatile and 1–15% non-volatile components [125]. The volatile constituents are a combination of monoterpene (limonene), sesquiterpene hydrocarbons and their oxygenated derivatives including: aldehydes (citral), ketones, acids, alcohols (linalool) and esters [126]. However, non-volatile portion of oil contains flavonoids that are deficient in flavonone glycosides but rich in hydrophobic flavone aglycons, particularly nobiletin [127,128]. Analysis of the hexane extracted oil showed extremely high concentrations of nobiletin (32%) and other flavone glycols [67].

## 5. In Vitro Antioxidant Potential

There are two classes of natural antioxidants, i.e., primary and secondary antioxidants [129]. Primary antioxidants, also called chain-breaking antioxidants, attack directly on lipid radicals and transform them into a stable form [130]. Secondary antioxidants, also known as preventive antioxidants, use different mechanism to slow down the rate of oxidation. The mode of action of primary antioxidants is the donation of a hydrogen atom [131]. On the other hand, secondary antioxidants employ different modes of action that include binding metal ions, absorption of ultra-violet radiation, decomposition of hydroperoxides, and scavenging oxygen [132]. Natural phenolic compounds found in plants can act as primary as well as secondary antioxidants through various mechanisms, and their activity can be accessed by monitoring the drop-off radical [133].

The antioxidant perspective of a compound varies with respect to the employed method when quantified through various antioxidants. Basically, two reaction processes are involved by virtue of that the citrus peel bioactive ingredient in the resultant extract triggers a protection mechanism against oxidation [134]. First, the single electron transfer method depends on the reduction of oxidation compounds like carbonyls, metals, and radicals [135].

Ghasemi, Ghasemi [136] evaluated the value of different total phenolic content in IC_50_ in citrus peels and reported values of 1.1, 1.4, and 2.1 mg/mL in *Citrus sinensis*, *Citrus limon*, and *Citrus paradis* peels, respectively. Later, Oboh and Ademosun [137] investigated the phenolic profiling of shaddock and grapefruit peel and observe that the free phenolics in shaddock peel were 6.5 mg/g, and in grapefruit peel, the amount of free phenolics was 13.1 mg/g, while the bound phenolics in grapefruit peel were 0.7 mg/g, and in orange peel, they were 6.8 mg/g. Nevertheless, grapefruit and orange peel have high free phenolic and bound phenolic contents, respectively. Moreover, free flavonoid content in shaddock peel and orange peel was 0.3 mg/g and 1.3 mg/g, respectively, but the bound flavonoid in grapefruit peel was 0.1 mg/g, and in shaddock peel, it was 0.4 mg/g; although, free flavonoids are higher in citrus fruits peel as compared to bound flavonoids. Phenolic compounds have Fe^2+^ chelating ability depending on the dose; nevertheless, free phenolics from orange peel had the highest chelating ability, that is, EC_50_ = 0.31 mg/mL, whereas bound phenolics had the least comparative chelating ability EC_50_ = 1.3 mg/mL [60]. Moreover, several studies have reported that antioxidants and radical scavenging active compounds are present in fruits, cereals, vegetables, and herb extracts [138].

The phenolics establish the chief portion of natural antioxidants existing in plants [139]. The supreme extensive and varied phenolics are flavonoids; they are secondary metabolites and broadly dispersed throughout the plant kingdom [140]. Antioxidants have extensive differences among several phenolic compounds, including the number of —OH groups, and the replacement by electron-donating alkyl groups of flavonoids raises the antioxidant prospective [141]. The phenolic contents in citrus peel are usually higher than in tissues, and the antioxidant potentials of several vegetables and fruits increase meaningfully with the increase of TPC [142].

DPPH is a reagent which is used to evaluate the free radical scavenging activity of antioxidants with an adsorption at 515–528 nm. It easily calculates samples in a short time and is too sensitive to identify very low-concentration ingredients and screen out the antiradical activity of several fruits and vegetables extracts [143]. Results have shown that *Citrus deliciosa* extract has free radical scavenging activity and prevents the initiation of chain reactions of free radicals by stabilizing several reactive species, with an IC_50_ value of 0.358 mg/mL [144]. Lim and Lim [145] reported that the IC_50_ concentration of *Citrus reticulata* Blanco mature peels and *Citrus reticulata* Blanco immature peels were 0.78 mg/mL and 0.46 mg/mL, respectively [145,146].

In another study, Khan, Abert-Vian [147] assessed the radical scavenging potential of orange peel by ultrasound-assisted extraction and solvent extraction. They concluded that using the sonication method, citrus peel potency to scavenge free radical increased by 30% in contrast to solvent extraction. Similarly, El-Aal and Halaweish [148] determined the antioxidant potential of two different citrus varieties. According to them, baladi peel extract had more DPPH activity as compared to navel orange peel extract, which ranged from 65 to 72% for both varieties.

In the ABTS method, a standard amount of Trolox was compared, and the relative scavenging ability of the radical in the aqueous phase was measured. The ABTS^+^ was produced by K_2_SO_4_, and it acted as brilliant medium for the estimation of the antioxidant activity of the hydrogen-donating and chain-breaking antioxidants. On dry weight basses, the TEAC value of extracts at 6 min reaction was 0.2 mmol Trolox Eq/100 g, and values of grapefruits peels was 7.31 μmol Trolox/g fresh weight [149]. Asghar, Khan [150] reported the values of various citrus species peel extracts like *Citrus aurantium* (16.19), *Citrus medica* (19.40), *Citrus paradise* (4.56), *Citrus sinensis* (7.21), and *Citrus aurantium* (1.28 mmol Trolox Eq/liter of extract).

Yu [151] determined the antioxidant activity of eight citrus peel flavonoids, two liminoids, and a coumarin by measuring the inhibitory power of bioactive components against linoleic acid oxidation. They concluded that flavonoids (54.1%) were stronger than limonoids and coumarin (<7%) in preventing the formation of an oxidized compound. This property was attributed to the aromatic ring structure of flavonoids. However Sultana, Anwar [152] quantified the antioxidant capacity of extracts from various agricultural by-products. They also interfered that citrus peel extract had 86.9% inhibition for linoleic acid oxidation.

Furthermore, Chatha, Hussain [153] has also determined antioxidant activity of various solvent extracts of grapefruits, lemon, and mussambi. The results’ interpretation has revealed that the maximum linoleic acid oxidation inhibition (91.78%) was recorded for 70% methanolic mussambi peel extract, while the minimum was recorded for 90% methanolic grapefruit peel extract (68.20%). Previously, Gursoy, Tepe [154] estimated the antioxidant properties of citrus peel oil by β-carotene and linoleic acid assay. They observed that citrus oil had a potency of 96.8 ± 0.2% to inhibit the oxidation of linoleic acid due to polyphenolic constituents.

Another method to evaluate the antioxidant strength of citrus peel is the β-carotene bleaching test that is dependent on the coupled oxidation of β-carotene as well as linoleic acid. In the absence of an antioxidant, β-carotene rapidly undergoes discoloration that reduces the absorbance of resultant material, detected spectrophotometrically. The main reason for this reduction is the coupled oxidation of β-carotene as well as linoleic acid that engenders free radicals, consequently bleaching out the orange color of β-carotene. However, in the presence of an antioxidant, the bleaching effect was hindered by neutralizing free radicals [155]. This main theme was elaborated by the findings of Gursoy, Tepe [154], who determined the antioxidant properties of orange peel oil. They analyzed that citrus oil has a power of 96.8 ± 0.2% to hinder linoleic acid oxidation due to its polyphenolic constituents.

A FRAP test was performed with a ferric tripyridyltriazine (Fe^3+^–TPTZ) compound and generated a colored ferrous tripyridyltriazine (Fe^2+^–TPTZ) that calculated the reducing power of antioxidants [156]. Normally, the reducing potential is related with those compounds can break by donating electrons [157]. Earlier, Xu, Ye [158] determined the heating and solvent effect on the antioxidant capacity of citrus peel. According to them, heating citrus peel extract at 120 °C for 90 min increased the Trolox antioxidant capacity from 19.66 to 33.14 mg Trolox equivalent antioxidant capacity (TEAC)/g of the dry peel weight. Ghafar, Prasad [159] reported that the FRAP value for *Citrus hystrix* samples was the highest at 89.0 ± 5.88, while a lower value of 48.18 ± 3.34 mg TEAC/g of dry peel weight was observed for *Citrus microcarpa* samples. The antioxidant potentials of citrus species were in the increasing order of *Citrus hystrix* > *Citrus aurantifolia* > *Citrus sinensis* > *Citrus microcarpa*. According to de Moraes Barros, de Castro Ferreira [160], who observed in vitro antioxidant capacity of various citrus parts, results relating to FRAP assay revealed that the antioxidant potential of peel extract was higher as compared to the pulp of the same fruit. Among five cultivars, mandarin peels had maximum ferric-reducing potential at 3897.9, rather than pulp at 744.0 ± 12.7 µmol of Trolox equivalent/100 g of fruit weight.

## 6. Citrus Peel-Enriched Nutraceutical Products

The pivotal connection between health and diet has increased the scope of diet based therapies against various physiological threats among the masses [161]. Due to the reason, the demand for such natural ingredients is increasing tremendously. By-products of the plant-based food industry are creating major disposal problems for concerned manufacturers, as well as for the environment, but due to their excellent nutritional profile, they can be utilized as a promising source of health-promoting compounds [162].

A large community of people relies on plant-based foods to fulfill dietary needs like carbohydrates, protein, fat, vitamin, and minerals. The development of novel food products is a multifarious and uncertain task that depends on scientific difficulty, consumer satisfaction, convenience, price, age and cultural habits [163]. Among them, cereals-based baked products and beverages occupy a central position for people of different age groups to satisfy their nutritional requirements [164]. Tarts, biscuits, cakes, and breads are more popular products consumed by a major segment of society due to their ready-to-eat nature, low cost, and availability in different shapes and flavor; also, these products are good at keeping quality and have a long shelf life [165]. Quality of the end product depends on the amount and nature of raw ingredients being used in the recipe. However, it was claimed that the inclusion of by-products of the citrus processing industry in baked products not only increases the nutritional attributes but also improves the sensory traits [166]. Baking is a complicated phenomenon that depends on chemical interaction of flour, with various product ingredients leading towards specific aromas, flavors, colors, tastes, and textural characteristics of chemical reactions occurring during whole process [167].

Breakfast is considered by nutritionists to be essential for learning and performance, especially for young children, but food preparation time in the mornings is usually limited [168]. Breakfast eaten in the car or in the office is continuing to grow, and portable hand-held foods have become the fastest-growing segment of the food industry [169]. While both carbohydrates and proteins are important energy sources at the start of the day, high-carbohydrate breakfasts (e.g., doughnuts, toaster pastries, and juice) are metabolized more quickly than a meal comprised of fruit, grains, and protein (e.g., orange juice, toast, eggs) [170]. The tart could be eaten without any preparation or could be warmed if desired [171]. Consumed with milk and fruit, a tart would provide a convenient, nutritious, high-quality protein meal with longer lasting satiety than a high carbohydrate breakfast [172]. Formulating, manufacturing, and marketing a product require a keen understanding of the target consumer and require testing, questioning, listening, and re-testing [173]. Consumers can really guide product development, and interaction with consumers should be initiated as early as possible and repeated at numerous occasions throughout the development stage [174]. The food industry will need to pay much greater attention to the scientific aspects of consumer needs and desires and to the potential for satisfying them through food technology [175,176].

One of the research groups, Nassar, AbdEl-Hamied [177], has reported that the addition of citrus by-products (peel) at different levels (5%, 15%, and 25%) affected the rheological aspects of resultant dough. As the level of peel increases, the water holding capacity is evaluated. This facet was attributed to the hydroxyl group of dietary fibers that the boost water absorption capacity of dough. Earlier, Sudha, Baskaran [178] explicated that the substitution of flour at different peel concentration contributed to dough stability that increased dough strength for longer time, but the mixing tolerance index declined as the protein content of citrus peel flour was low.

Sugar is another main ingredient that affects the taste, color, and texture of end product [179]. If used in excess, it decreases the viscosity of dough and increases cohesion that causes the spreading of biscuits and browning of cake crust, as well as hardening of products [180]. Therefore, the exact amount is very important as it helps to retain water that will cause the spreading of proteins and carbohydrates, thus developing a crispy texture in biscuits and causing the softening of cakes. There are demands for sugar-free bakery products that have low calorific value. In this respect, low-calorie artificial sweeteners are being used to make dietetics and diabetic bakery products, e.g., sorbitol, fructose, and mannitol [181,182]. They are basically polyols (mannitol and sorbitol) that are not completely metabolized in human body as a result contributes less energy and, ultimately, the blood glucose level does not rise rapidly [183]. But their sweetness is more than for the same quantity of sucrose. Results related to the acceptability of cake have shown that replacing 50% sucrose with fructose improves the texture, color, flavor, and softening of the crumb [184]. Similarly, in the case of biscuits prepared with sucrose and fructose (50:50), products have better sensory attributes up to a two-month storage period [185].

In bakery products, the fundamental role of shortening is to provide a lubricating effect and flexibility to dough. In the case of biscuits and cakes, highly elastic dough is not required as it leads towards the hardening and shrinkage of the end product, which is not desirable [186]. Accordingly, the exact amount of shortening is very important as it has a pronounced effect on dough machinability throughout the whole process, as well as the texture and sensory attributes of the end product after baking [187]. The present rate of mortality due to cardiovascular diseases is mounting each day, and a decrease in serum HDL cholesterol level is a basic indicator of heart diseases [188]. Scientific research has proved that dietary fat has a pronounced effect on serum total cholesterol, LDL, and HDL levels [189]. Subsequently, the type and quantity of fat being used in bakery products should be taken into account [190]. Earlier findings have elucidated that saturated and trans fatty acids tend to increase serum total cholesterol as well as LDL, while the HDL level decreases [191]. On the other hand, polyunsaturated fats boost the serum HDL level with a significant reduction in total cholesterol and serum LDL level [192]. In bakery products, substituting saturated fats up to 5% of energy with polyunsaturated fats rather than decreasing their quantity has a potential towards serum LDL reduction by 0.26 mmol/L [193,194].

Sensory evaluation is a cardinal step to assess the overall acceptability of end products through a trained panelist [195]. Various attributes like color, flavor, texture, taste, mouth feel, and acceptability were determined using a hedonic structured scale [196]. Keeping the quality of baked goods is very important as it not only affects the nutritional quality but also the economic value [197]. An important factor that must be taken into consideration is the rancidity of fats and oils that effect the taste and flavor of a product if stored for longer time [198]. To overcome this problem, antioxidants are being used to prevent the oxidation of fats [199]. Synthetic antioxidants are now restricted in various food items due to their carcinogenic effects [200].

In this respect, Magda, Awad [201] evaluated mandarin and navel orange peel as sources of antioxidant compounds, along with their contributions as a source of fiber, minerals, and coloring and flavoring agents. They supplemented wheat flour at three different concentrations (5%, 10%, and 15%) for hard biscuits’ formulation. From the whole trial, it was concluded that replacement up to 5 and 10% not only improved sensory attributes but also decreased lipid oxidation, as it was indicated from the peroxide values of mandarin (12.5 meq/kg fat) and navel orange peel biscuits (10.3 meq/kg of fat) as compared to the control (35 meq/kg fat) when they were stored for 6 months at 25 °C.

Keeping in view the above advantages of peel powder and extracts, their utilization in food products in order to prevent lipid peroxidation, prolong shelf life and improve organoleptic properties is very economical. Although they must be added in larger amounts as compared to synthetic chemicals, there is no legislation regarding to their dosage level in food products.

## 7. Citrus Peels Current Uses

**Figure 4:**
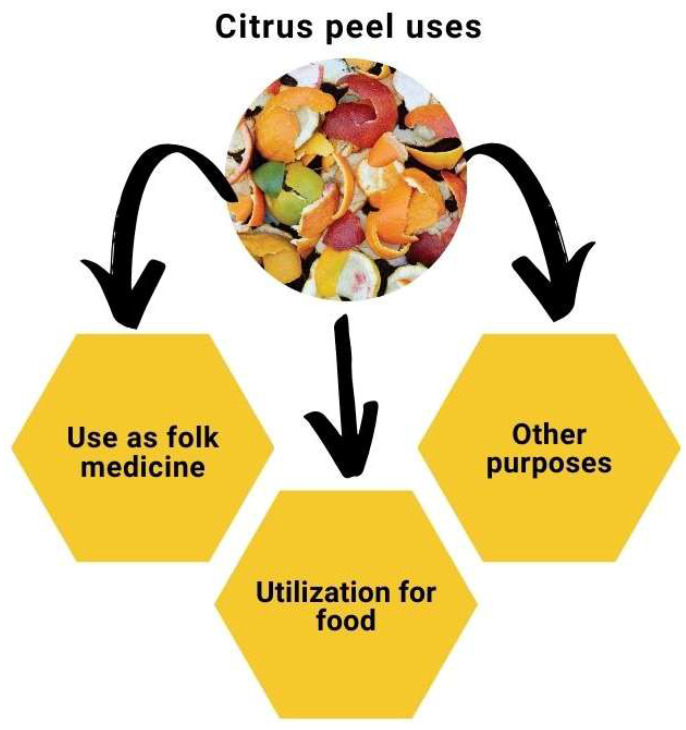
Uses of citrus peel waste.

Citrus peels from a variety of species have been utilized in pharmaceutical and non-pharmaceutical uses such as ethanol and methane generation, as well as food components [202]. It is reassuring to find that the peels, a food waste and by-product of citrus fruits, have significant potential for application, as displayed in Figure 4 [203]. In food, they serve as natural preservatives, colorants, and sources of dietary fiber, enhancing nutritional properties and stability. For example, citrus peel extracts can be used as natural antioxidants in food and cosmetic products [204]. Citrus peel waste can be converted into value-added products like bioenergy sources and edible packaging materials, offering eco-friendly alternatives to traditional materials. Leveraging these by-products not only fosters product innovation but also aligns with sustainable waste management practices, contributing to a circular economy and resource efficiency. Citrus waste valorization represents a holistic solution to waste management challenges, environmental sustainability, and circular bio economy goals.

### 7.1. Use as Folk Medicine

In Japan, dried peels from mature citrus fruits are used as crude medicine “Chimpi” and “Touhi” [205]. In a similar way, Citri Reticulatae Pericarpium (CRP), the dried and ripened citrus peel, is a well-known traditional Chinese medicine that has been widely utilized as a food and supplements in China [206]. CRP, a key Chinese herbal medication, has been extensively used for thousands of years to treat respiratory and digestive disorders such as bronchitis and asthma. In the folk tradition of southern China, CRP is also commonly used as a healthcare food in southern China, where it is cooked with meat for soup, mixed with beans and rice to make porridge, or manufactured into different snacks [203]. Many CRP varieties are found throughout China, including Guangchenpi (Citrus reticulata ‘Chachi’), Dahongpao (Citrus reticulata ‘Dahongpao’), Wenzhou migan (Citrus reticulate ‘Unshiu’), and Fuju (Citrus reticulate ‘Tangerina’), as documented in the People’s Republic of China Pharmacopoeia [203]. In general, Guangchenpi is classified as a geoherb because of its superior quality, and the extracts have revealed the highest level of polymethoxyflavones coupled with the strongest antioxidant and anti-inflammatory properties [207]. Many factors influence the overall quality of CRP, including varieties, manufacturing, and storage periods, with the storage time having the greatest impact due to chemical component conversions or content variations [206]. Traditional folk wisdom states that the longer the storage duration is, the greater the CRP impact will be. Yet, subsequent research has indicated that the metabolites in CRP are initially elevated and then reduced with an increase of storage duration [208].

With subsequent clinical research, the usage of CRP has expanded beyond the respiratory and gastrointestinal tracts to include cardiovascular, anti-tumor, anti-oxidation, and anti-inflammatory properties [209]. But there is still a paucity of comprehensive studies on the pharmacological applications of CRP, and its method of action remains to be researched [203].

### 7.2. Utilization for Food and Other Purposes

Citrus peels have few non-pharmaceutical applications and are now used for:

#### 7.2.1. Extraction of Pectin

The global demand for pectin is around 40,000 tons per year, with the United States (6500 tons), Russia (2700 tons), and Japan (2300 tons) being the primary consumers. Citrus peel pectin, the most common source of commercial pectin manufacturing with an annual production of 124 million tons, is widely utilized as a gelling agent, emulsifier, and fat alternative in the food industry sector [203,210].

#### 7.2.2. Livestock Feed

Citrus peels have been used as cattle food as well as a health supplement for animals due to their high nutritional value [211]. According to studies, ruminant and non-ruminant feed costs can be decreased without polluting the environment. Nonetheless, the end product comprises of relatively poor animal feed, due to the low protein level and high quantity of carbohydrates, which are important limitations towards the use of feed made entirely of citrus peels [203,212].

#### 7.2.3. Food Products

Citrus peels are commonly utilized in the production of a variety of food items. They are typically consumed as a raw material for the production of baked foods, jams, and pickled fruits around the world. Citrus peels are used in Indian dishes, including custards, curries, and gravies, to improve their flavor and odor. In Europe, they are frequently utilized in sweet delights [213]. It is documented that lemon peel has been used to cooked and dry-cured sausages, with outstanding results [214]. Surprisingly, the inclusion of pomelo (Citrus grandis Osbeck) peel can enhance the fiber amount in rice noodles [215].

## 8. Physiological Threats

**Figure 5:**
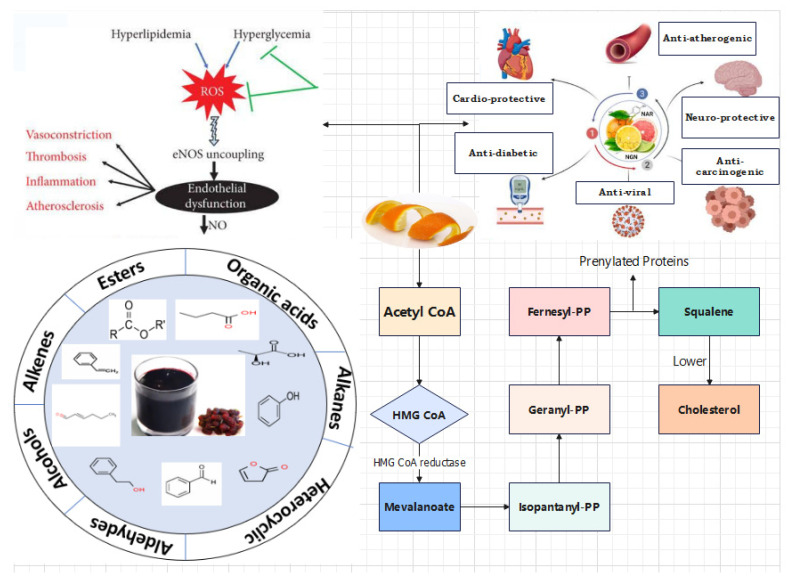
Therapeutic effects of citrus peel waste.

**Figure 6:**
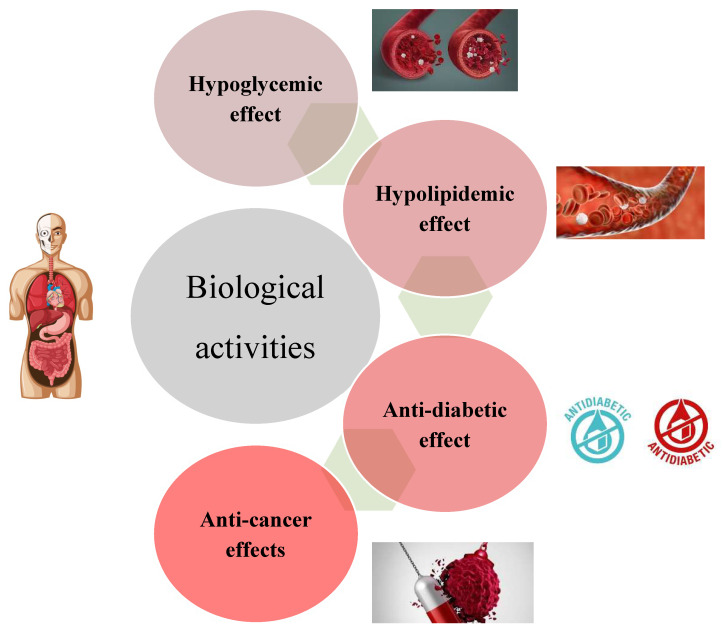
Biological activity of citrus peel waste.

Diet-based treatments are being administered both in developed and developing countries for health alleviation due to their inescapable biological safety [216,217]. However, with the onset of the 19th century, the modern drug therapy has outshined the thought of “Food as medicine”, making this concept much more insignificant [218]. Lately, in the 1900s, the trend once again drifted towards diet-based therapy against disease prevention and towards health promotion [219,220]. Polymethoxylated flavones (PMF) are distinctive group of flavonoids that are solely present in citrus fruits, especially in orange and mandarin peels [221]. Polymethoxylated flavones have a wide spectrum of therapeutic effects like antioxidant potential, antithrombiotic properties, chemopreventive action, and cholesterol-lowering effects, as illustrated in Figure 5 and Figure 6 [59,222]. Earlier studies have elaborated on their chemopreventive attributes against the biosynthesis of adhesion molecules, the appearance of tumor factor-R (TNFR), the spread of tumor to surrounding animal tissues by enhancing apoptosis, and the minimization lymphocytes propagation and platelet aggregation. They are more permeable and readily absorbed via the small intestine into blood circulation due to their lipophilic nature [223].

Citrus peel is a waste material for one industry, but it is a useful ingredient for other industries because their proper utilization in the pharmaceutical and nutraceutical industries will not only offer a potential for cost-effective therapeutics but also enhance the value of functional and nutraceutical food [29,51]. There is a need to create awareness among people to make use of such a phytochemical-rich diet that not only fulfills their dietary needs as but also normalizes body physiological functions because to enjoy good health is right of every person [224].

Health investigations on citrus polyphenol and phenolics have focused completely on their flavonoid constituents, although hydroxycinnanamates do happen in fruits [112]. Four types of flavonoids are present in citrus: flavonols, flavanones, flavones, and anthocyanins (present only in blood oranges) [65]. The dominating parts of citrus fruits are flavones and flavanones, which have distinct properties, while other two, anthocyanins and flavonols, are widely present in many other species [225,226].

### 8.1. Hypoglycemic Prospective

Asia and Africa are most affected areas where diabetes mellitus has been increasing at a rate of two- to three-fold [227]. Bioflavonoids and dietary antioxidants offer safety against the expansion of diabetic problems [228]. The relation between the intake of flavonoids and the subsequent incidence of numerous long-lasting diseases like diabetes mellitus has been evaluated [229]. Various drugs have been recommended to control blood glucose levels for hyperglycemic people, but they are less effective and required in large quantities, and some of them are toxic [230]. One of the cost-effective and readily available sources to treat diabetic patient is to take flavonoid-rich foods due to their antioxidant properties and hypoglycemic potential [231]. Citrus peel exhibits a heterogeneous group of flavonoids, having a broad spectrum of biological activities against cardiovascular disorders, cancer, diabetes, hypercholesterolemia, and oxidative stress. The main bioactive components of citrus peel, hesperidin and naringin, have antioxidant potential at the early stages of diabetes mellitus and for its associated complications [25,59].

*Citrus medica* extracts show hypoglycemic potential in α-glucosidase and α-amylase inhibition assays [232]. In a study, glucose freely absorbed from the gastrointestinal tract into the blood after the breakdown of glycosidic bonds in carbohydrate-based digestible food contained α-glucosidase and α-amylase. These enzymes’ inhibition resembles the reduction in the peaks of high blood glucose in diabetics [233]. Hesperetin and qurecetin both inhibit the activity of α-glucosidase from bread yeast (*Saccharomyces cerevisiae*) at about 50 and 7 l M in IC_50_ values, respectively [234]. *Citrus medica* n-hexane extract inhibits the activity of a-amylase 625 µg/mL in IC_50_ [235,236]. *Citrus medica* essential oil also showed anti-diabetic and hypoglycemic activity in in vivo studies [237]. This activity was related to the phytochemicals that are present in peel oil, which might facilitate access to the enzymatic site [238,239].

Tundis, Loizzo [123] determined the in vitro hypoglycemic activity of citrus peel extract by the α-amylase and α-glucosidase inhibition method. Orange peel extract was reticent to both α-amylase and α-glucosidase, having IC_50_ values of 258.7 and 263.2 µg/mL, respectively. These results are five- to seven-fold less than acarbose-treated patients, for which the IC_50_ value was 50.0 µg/mL for α-amylase and 35.5 µg/mL for α-glucosidase.

Hypoglycemic agents from plants sources ensure keen attention in inhibiting diabetic problems; meanwhile, natural sources are normally considered to have fewer side effects, with fewer being moderately toxic compared to artificial medicines [240]. The oral administration of hesperidin has a late effect on the motherly glucose level [241]. Certainly, before organogenesis. that is, 1–7 days, hyperglycemia was, to some extent, reduced [242]. Hesperidin is a major glycosylated flavonones most commonly present in grapefruit, orange, and lemon fruit. Previous outcomes have revealed that hesperidin, naringin, and rutin, each at dose level of 0.05% of diet, reduced blood glucose levels by 18%, 16%, and 21%, respectively, in diabetic rats induced by streptozotocin [243]. At the same time, daily oral administration of these bioflavonoids to humans at levels of 5, 10, and 15 mg/kg of body weight significantly decreased blood glucose levels by 17%, 23%, and 33%, respectively [244].

In a recent study, Toumi, Merzoug [245] concluded that providing a hesperidin-based daily diet to pregnant mice imitated the capability of natural flavanone modulate parental weight, improving maternal hypoglycemic activity. This possible anti-teratogenic effect is worthy and a clear hidden prophylactic outcome of hesperidin against diabetics.

Hesperidin has anti-diabetic and anti-teratogenic effects in pregnant diabetic mice;some flavonoids, predominantly hesperidin and quercetin, have been involved in several studies and have qualified antioxidant and hypoglycemic effects [246]. Frequent supplementation via oral administration maintained a high concentration of polyphenols in blood plasma; although the dose used was very low, hesperidin exerted continuing results due to the flavanones’ own high bioavailability [247].

Hesperidin-treated diabetic mice had no effect on skeletal dysmorphogenesis; hyperglycemic activity was directly involved in skeleton mal development and protected glucotoxicity-targeted developed bones [248]. In the dysmorphogenesis of a progeny subject to skeletal and visceral from diabetic animals, the susceptibility to these morbid proceedings has been shown to be strongly connected to strain’s genetic background [249].

A study on hesperidin supplementation for 35 consecutive days showed a significant elevation of induced diabetes in the blood glucose level; almost 15% of diabetic animals returned to the normal level of blood glucose, and other animals showed an almost 53% reduction in the blood glucose level, in comparison to the diabetic control group [250]. Hesperidin holds a significant part in inhibiting the development of hyperglycemia, partially enhancing glycogen synthesis and hepatic glycolysis by dropping liver gluconeogenesis [248,251].

An in vivo study on the effect of citrus peel extract (300 and 600 mg/kg) on diabetic rats showed a significant reduction in blood glucose levels by controlling the glucose regulatory enzymes of the body [252]. Chronic oral administration of peel extract (100–600 mg/kg/day) to rats for 30 days maintained blood glucose levels and mitigated the progression of liver dysfunction caused by diabetes, even after taking a high-glucose diet that revealed its glucose tolerance potential in hyperglycemic rats [253].

In vitro and in vivo studies have depicted that hyperglycemic disorder raises oxidative stress and weakens body intracellular antioxidant resistance mechanism that increase the risk of type 2 diabetes. Inclusion of flavonoids-rich food items in daily dietary plans is a leading step towards a healthy lifestyle [254]. In this respect, taking citrus peel-supplemented food products and its various preparations endows the body with natural antioxidants that have strong free radical scavenging activity [59]. This not only protects β cells from oxidative damage but also chelates metal ions like copper and iron that are the leading causes of cancer and cardiovascular complications [255].

### 8.2. Hypolipidemic Potential

Cardiovascular diseases are the major leading cause of morbidity and mortality all over the world. High cholesterol and oxidation of LDL triggers the events leading to starting of atherosclerosis [256]. A defective immune system results in the initiation of various health disparities, categorized as autoimmune disorders and immune [257]. Dietary nutritional status has a significant effect on the antioxidant immune system of the body and deficiency of certain nutrients; for example, vitamin E, selenium, and polyphenols increase the diseased status of oxidative stress, diabetes mellitus, and atherosclerosis [2,258]. Functional foods or their functionally active molecules have shown therapeutic potential, containing antioxidant, anti-inflammatory, anti-cancer, and immunomodulation effects [259,260].

Scientific research has confirmed that citrus peel-derived flavonoids’ intake ranges from 2.6 to 68.2 mg/day and has the potential to alleviate mortality rate due to cardiovascular disorders in Japan [261]. An in vivo study reported that the cholesterol level decreases due to citrus flavonoids. Citrus peel flavonoids were found to be beneficial in reducing the metabolism of acetylated LDL (acLDL), modifying lipoprotein involved in macrophage foam cell formation, and binding with SR-A that expresses on cultured macrophages and is involved in cholesterol accumulation [262].

An in vitro study relating to the cholesterol-lowering potential of both hesperidin and naringin on hepatoma HepG2 cells of the human body confirmed that citrus peel bioflavonoids have the potential to reduce secretion of LDL-linked apolipoprotien B (apo B), as well as decrease the IC_50_ value for apo B (concentration required for 50% reduction of apo B). It was reported that the IC_50_ concentration was 142 and 178 µM for hesperidin and naringin, respectively. Basically, hepatoma HepG2 cells were used in this study as they are involved in the synthesis and catabolism of apo B, having lipoproteins such as LDL and VLDL [263]. Nobiliten is polymethoxylated flavone that is mostly found in orange peel and is effective against inflammation, cancer, and hyperlipidemia and provides neuroprotective effects in Alzheimer’s disease. In rats study, 0.1% nobiletin in their diet reduced white adipose tissue, with a significant increase in HDL and apolipoprotien A-1 without modifying triglyceride level [264]. These findings have justified the hypolipidemic effect of nobiletin under hypercholesterolemic conditions [264].

In the positive control hypercholesterolemic group, a significant raise in serum triglyceride, total cholesterol, very low-density lipoprotein, low-density lipoprotein, and artherogenic index was observed in comparison to the negative control group. When the negative control group was administrated 1% methanolic extract of citrus peel, it resulted in a reduction of the total cholesterol [265].

Supplementation of diet with 5% orange peel powder and 1% orange peel extract decreased body weight ratio; this was compatible with previous outcomes where the body weights of a citrus peel-supplemented rat group increased during a trial of 28 days. Concluding data have revealed that orange peel extract was more effective in reducing serum total cholesterol and LDL, VLDL, and triglyceride but had a positive impact on HDL [266,267].

Scientific research has exposed defect in body defense system and formation of reactive oxygen species (ROS) among diabetics, leading towards other complications such as hypertriglyceridemia and hypercholesterolemia. Oxidative stress due to diabetes causes oxidation of LDL that was facilitated by 15-lipoxygenase (15-LO) present in the liver [268]. This will make hard plaque in the blood vessels and increase the risk for atherosclerosis and stroke. Under this condition, the HDL level decreases that are involved in the removal of excess cholesterol from atherosclerosis plaque occurs, thus maximizing chances for cardiovascular disorders [269]. However, citrus peel flavonoids, especially flavonones, flavones, and flovonol, have strong inhibitory effects for 15-LO enzyme [59]. The inhibitory potential of flavonones (hesperidin) is higher as compared to flavones (nobiletin), as the IC_50_ value for hesperidin is 180 µM that is much higher than for nobiletin (86 µM) due to the presence of a methoxy group at the third position [247,270].

Moreover, serum total cholesterol, low-density lipoprotein, and artherogenic index was reduced in rats treated with methanolic extract of citrus peel, and a significant increase in high-density lipoprotein in all groups in comparison to the control group was observed [271,272]. Supplementation of hesperidin and naringin mixture along with grapefruit peel extract had a hepatic triglyceride- and hepatic cholesterol-lowering effect. Nevertheless, the relations of high-density lipoprotein to total cholesterol remained higher in the positive control group, while conflicting results were observed for the atherogenic index [273,274].

### 8.3. Anti-Diabetic Effects

Type 2 diabetes is a degenerative condition that is increasingly spreading across every age category. It is defined by impaired metabolism of glucose, increased levels of carbohydrate hydrolyzing enzymes, resistance to insulin, and pancreatic beta cell malfunction and destruction [275]. One of the most frequent pharmacological methods to type 2 diabetes management is to regulate blood glucose levels by inhibiting carbohydrate hydrolyzing enzymes. Enzymes like α-amylase and α-glucosidase are good biomarkers to prevent hyperglycemia [276]. These enzymes have a role in the degradation of starch to glucose, which increases the uptake of glucose into the bloodstream and causes hyperglycemia in diabetic individuals [277]. Yet, the reduction of these enzymes reduces glucose absorption and lowers postprandial hyperglycemia [278]. These enzymes are targets for artificial hypoglycemic drugs like acarbose and voglibose. Yet, the usage of these medications is limited due to their negative effects [279]. Lim and Loh [280] examined the in vitro inhibiting activity of phenolic extracts from *Citrus maxima, Citrus hystrix, Citrus aurantifolia* and *Citrus microcarpa* peels. *Citrus microcarpa* peel phenolic extracts showed the best inhibitory activity against α-amylase and α-glucosidase. Citrus peels may inhibit αamylase and α-glucosidase activity in vitro due to their high phenolic content. Citrus flavonoids can lower glucose levels in diabetic individuals by inhibiting α-amylase and α-glucosidase effects. Rutin, quercetin, quercetrin, luteolin, and kaempferol block α-amylase and α-glucosidase activity by forming hydrogen chains with the amino acid sequences at their binding site [281,282].

Xiao, Ni [282] found that hyroxylations and non-glycosylations of flavonoids enhanced their inhibitory influence on α-amylase performance. Tadera, Minami [283] expounded that quercetin, apigenin, naringenin, kaempferol, and luteolin effectively block αamylase performance through their hydroxyl groups and ring geometry. Citrus peels include phenolic acids like caffeic, gallic acids, chlorogenic, ferulic, and p-coumaric, which can inhibit α-amylase enzyme functioning [112,284,285]. Pereira, Cazarolli [286] found that flavonoids like rutin, kaempferol, and quercetin reduced the production of intestinal α-glucosidase. Muhtadi, Azizah [287] also found that a high dose of *C. sinensis* peels (500 mg/kg) lowered blood glucose levels in diabetic mice. In a similar way, *Citrus limon* demonstrated a glucose-lowering effect identical to a conventional medication (glimepiride) in alloxan-induced diabetic mice [288].

Parkar and Addepalli [289] found that *Citrus sinensis* peel extract (100 mg/kg and 200 mg/kg) reduced diabetic nephropathy in streptozotocin-induced diabetic rat models. Diabetes-related nephropathy is defined by the buildup of extracellular matrix debris in renal cells. The peel extract’s antidiabetic action was not connected with a decrease in plasma glucose, but rather with a decrease in renal levels of collagen caused by suppression of Gelatinase A (MMP-2) and Gelatinase B (MMP-9). Gelatinase A and B are metalloproteinases (MMPs) that can degrade proteinaceous constituents of the matrix outside of cells. The elevation of these enzymes has been linked to renal disorders, particularly nephropathy caused by diabetes. The peels of citrus have also been demonstrated to aid in healing of wounds in diabetic patients. Ahmad, Ansari [290] evaluated the efficacy of oral therapy with *Citrus limon*, *Citrus paradise*, and *Citrus sinensis* peel extracts on diabetic rat skin wound healing. The peel extracts lower blood sugar levels, promote wound healing through tissue growth, and stimulate collagen formation [291].

### 8.4. Anti-Oncogenic Effects

Cancer cells have several distinguishing characteristics, notably the lack of angiogenesis, apoptosis, and metastasis [292]. Several therapeutic treatments in the treatment of malignancies have addressed the systems that regulate the inhibition of apoptosis, angiogenesis and metastasis [293]. Some beneficial substances induce apoptosis by inhibiting proteasome activity [294,295]. *Citrus paradisi*, *Citrus sinensis,* and *Citrus maxima* peel phenolic-rich extracts have been demonstrated to suppress proteasome function in primary (Caco-2) and metastatic (LoVo and LoVo/ADR) colon cancer cells in a concentration-dependent way [296,297]. As malignancies develop in size, more vasculature is necessary to support development [298]. As a result, cancers secrete vascular endothelial growth factor (VEGF), which stimulates the growth of blood vessels within the tumors [299]. Angiogenesis is a process that is necessary for tumor development and metastasis [300]. Thus, blockage of VEGF or its receptors has grown into targets for therapies to avoid vascularization [301]. Pan, Li [302] demonstrated that mixed citrus peel extracts lowered VEGF levels of proteins and inhibited skin-related inflammation indicators in an animal experiment.

Metastasis is a vital step in all forms of cancer. The group of enzymes known as metalloproteinases (MMPs) is a prominent therapeutic focus for metastasis prevention. Cancer cells manufacture MMPs to penetrate the tissues around them [303,304]. Free and bound phenolic extracts from *Citrus sinensis* peels reduced MMP activity in colon cancer cells (LoVo, Caco-2 and LoVo/ADR) [305]. In addition, an extract of various types of citrus peels reduced the production of VEGF and MMP-9 in rat colon tissues. The inflammation has been linked to molecular alterations that lead to the growth of several cancer forms [306]. Overexpression of inflammatory enzymes like inducible nitric oxide synthase (iNOS) and cyclooxygenase-2 (COX-2) promotes cancer and aberrant cell proliferation [307]. Suzawa, Guo [308] demonstrated that a blend of citrus peel extracts reduced iNOS and COX-2 expression in azoxymethane-induced discomfort of the mouse colon. The chemopreventive characteristics of ethanolic extracts of *Citrus reticulata* and *Citrus aurantifola* peels have been developed through their ability to trigger apoptosis [309], reduce carcinogenesis [310], prevent new blood vessel development [311], and improve cytotoxicity of other agents used for chemotherapy [305].

Citrus peels’ anticancer activities are strongly tied to their phenolic component, particularly flavonoids [112]. Tangeretin, a polymethoxyflavonoid derived from citrus fruits, caused apoptosis in breast carcinoma (MDA-MB-435 and MCF7), cancer of the colon (HT-29) and leukemia (HL-60) cells [312,313]. Citrus peels contain an excessive amount of nobiletin, which has anticancer characteristics because it induces apoptosis via cell cycle control [314]. Morley, Ferguson [315] found that it enhanced the cytotoxicity of doxorubicin in MCF-7 and T47D cells. The anticancer activities of hesperidin, hesperetin, and naringin, which are plentiful in citrus peels, have also been investigated [270]. The flavonoids involved were proved to up-regulate p53 and caspace-3 in MCF-7 and HL-60 cells to cause apoptosis, control cell cycle by suppressing CDK2 and p21 activity [310,316]. Ellagic acid and luteolin were demonstrated to suppress VEGF in endothelial cells [317]. Anthocyanins suppressed MMP-2 and MMP-9 expression in fribrocarsoma cells and HT-29 cells [291,318]. Citrus peels have proven excellent anticancer properties. However, additional in vivo research and clinical studies are encouraged.

## 9. Citrus Peel-Based Food Products Accessible on the Market

**Figure 7:**
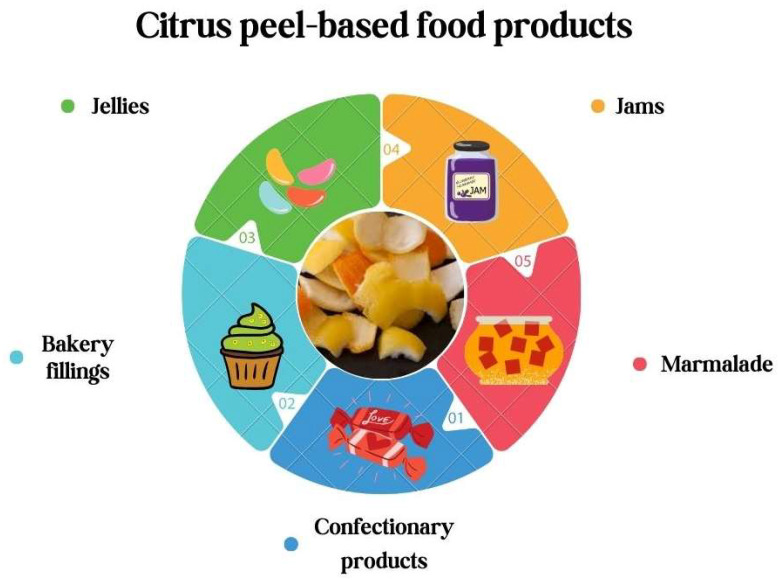
Citrus peel-based food products in market.

The advantageous effects of citrus peel-based uses in the food industry have been recognized around the world. As a consequence of the well-documented health benefits, numerous food sectors have launched initiatives to develop citrus peel-based food products, as displayed in Figure 7 [319]. Citrus peel-based teas are very popular in the worldwide food industry [320]. Nowadays, there are several tea brands on the market that properly mix citrus peel. Certain products utilize orange peel oil-based granules and orange peel oils [134,319]. Furthermore, orange peel comprising dark chocolate, made from freeze-dried orange peel and orange oil, is accessible on the market [321]. Citrus essential oils, on the other hand, have been on the market for a long time [322]. Numerous essential oils on the market are used as flavoring compounds in numerous food products and for consumption directly after adequate dilution [323]. In addition, food products, such as dried citrus peel particles, designed specifically for culinary use, are available on the market. Furthermore, citrus peel pectin is commonly used as a source of dietary fiber [324]. There are several dietary supplements on the marketplace that include pectin. Among the most well-known citrus peel-based food items on the market is frozen citrus peel pieces, which offer an aromatic touch to the foods [37].

## 10. Future Perspectives

The use of citrus peel in food industry applications has become an important research subject as the world seeks environmentally friendly alternatives to waste production in many industries [37]. Innovative green extraction methods are commonly used to extract bioactive components from the peels of citrus fruits [325]. Many studies have investigated the effectiveness of supercritical fluid, ultrasound-assisted and microwave-assisted extraction, as well as environmentally friendly techniques for extracting bioactive substances from peels of citrus fruits [326,327]. Still, the food industry faces a hurdle in guaranteeing the stability of extracted substances for commercial use [328]. Several studies have been conducted to investigate the encapsulation of bioactive substances [329]. Nano-encapsulation is now a prominent goal in future research. Furthermore, numerous studies have used pectin derived from citrus peels as a barrier material for covering bioactive substances [330]. More research is needed to determine how to successfully use pectin to nano-encapsulated bioactive substances [331]. On the other hand, the trend is to create emulsion-based systems for delivery with hydrosoluble and liposoluble (citrus oil) materials to effectively use citrus-based components [332]. In contrast, as technology improves, there is an urgent need to target research towards possible fourth industrial uses that focus on the intelligent production of functional food and customized diets using 3D food printing techniques. Consequently, the bioactive components and pigments derived from citrus peels can be productively exploited to create various kinds of inks for food printing in 3D [319].

## Figures and Tables

**Table 1:** Compositional profile of citrus peel.

Components	Chemical Composition	References
Total sugar content	165 mg/g	[58,59,60]
Pectin	128 mg/g	
Crude fiber	86 mg/g	
Crude protein	42 mg/g	
Lignin	22 mg/g	
Total ash	21 mg/g	
Crude fat	15 mg/g	
Carbohydrate	715.7 mg/g	
Phenolic compounds	179 mg/g	
Vitamin C	65 mg/g	
β-carotene on dry weight basis	0.021 mg/g	
Hesperidin	0.066 to 66 mg/g	
Narirutin	0.03 to 26.5 mg/g	

## Data Availability

The original contributions presented in the study are included in the article, further inquiries can be directed to the corresponding author.
